# The amount and chemistry of acylsugars affects sweetpotato whitefly (*Bemisia tabaci*) oviposition and development, and tomato yellow leaf curl virus incidence, in field grown tomato plants

**DOI:** 10.1371/journal.pone.0275112

**Published:** 2023-11-27

**Authors:** John R. Smeda, Hugh A. Smith, Martha A. Mutschler

**Affiliations:** 1 Entomology and Nematology Department, University of Florida, Gulf Coast Research and Education Center, Wimauma, Florida, United States of America; 2 Plant Breeding and Genetics Section, School of Integrative Plant Science, Cornell University, Ithaca, New York, United States of America; Jeju National University, REPUBLIC OF KOREA

## Abstract

The objectives of this study were to ascertain the impact of endogenous production of trichome-exuded acylsugars on insects and insect transmitted virus by evaluating tomato lines and their hybrids bred for acylsugar production under field settings on whiteflies and the whitefly-transmitted tomato yellow leaf curl virus. Specifically, we utilized a diverse array of tomato lines and hybrids bred for changes in acylsugar amount or type, grown in three field trials under natural whitefly and virus pressure, to investigate whether the amount of accumulated acylsugars and or the chemical profile of the acylsugars were associated with greater resistance to whiteflies and reduced incidence of tomato yellow leaf curl virus. There was considerable variation in the abundance of whitefly eggs and nymphs and incidence of tomato yellow leaf curl virus across experiments and between entries. Increasing amount of acylsugars accumulated by the tomato entries was associated with a reduction in the abundance of whitefly eggs and nymphs and a reduction in the incidence of tomato yellow leaf curl virus. Additionally, we identified lines with changes in several acylsugar fatty acids that were associated with decreased abundance of whitefly eggs and nymphs and reduced incidence of tomato yellow leaf curl virus. These results inform the utility of acylsugars as a host plant defense system for improving resistance to whiteflies and their transmitted viruses, with potential for reducing insecticides as a control method for whiteflies and provide breeding targets for optimization of existing acylsugar tomato lines to create lines with the most efficacious amount and chemistry of acylsugars.

## Introduction

Tomato is a widely cultivated and important vegetable crop that suffers from a wide variety of pests and pathogens. One of the most pervasive, invasive and damaging pests worldwide is the sweetpotato whitefly (hereafter abbreviated as “whitefly”) *Bemisia tabaci* MEAM1 (Gennadius) (Hemiptera: Aleyrodidae), which is a member of the cryptic *Bemisia tabaci* species complex [1]. This polyphagous whitefly threatens tomato production directly through feeding damage that can diminish marketable yields, but most importantly through the transmission of over 300 plant viruses [2], including the most widespread and damaging insect-transmitted tomato disease, *Tomato yellow leaf curl virus* (TYLCV) a begomovirus of the *Geminiviridae* family [3–6]. Management of whiteflies and TYLCV involves clean culture (starting with virus-free transplants and promptly destroying harvested fields), planting into metalized plastic mulches to repel the vector, deploying TYLCV-tolerant tomato varieties when appropriate, and intensive insecticide use [4, 7]. When whitefly density and virus incidence are high, even weekly applications of insecticides do not provide adequate control [7–9]. Whiteflie*s* have developed resistance to many classes of insecticides in different regions of the globe [10]. Alternatives to chemical control of whiteflies are needed because of inconsistent results and resistance concerns associated with insecticides, and potential negative impacts of insecticides on the environment, non-target organisms including pollinators, and human health. [11–16]. A promising option for reduced reliance on insecticides is the identification of virus and insect resistance in wild relatives of tomato and their transfer to cultivated tomato. Despite typical reproductive barriers, several genes mediating resistance to TYLCV have been identified in wild relatives and introgressed into tomato [17–28]. While these TYLCV resistance genes are the most effective strategy for control of whitefly-transmitted viruses like TYLCV currently, strains of TYLCV that can overcome these genes have already been identified and are being transmitted by whiteflies [29–32].

Acylsugars are a class of secondary metabolites that have consistently been associated with resistance to arthropods and are found in numerous wild *Solanum* species [33–47]. In *Solanum pennellii* and derived populations, acylsugars have been associated with resistance to several species of whiteflies including the sweetpotato whitefly [42, 48–55] and are secreted from type I/IV trichomes. Acylsugars are composed of a sugar backbone, either sucrose (resulting in acylsucroses) or glucose (resulting in acylglucoses), to which are esterified several aliphatic acids, ranging from 4 to 14 carbons in length. These fatty acid acyl chains can be straight-chained or branched (iso or anteiso) [55–63]. Cultivated tomato accumulates trace amounts of acylsugars due to missing genetic loci necessary for either glandular trichome production or acylsugar biosynthesis/secretion. *Solanum pennellii* accessions exhibit an immense diversity of acylsugars [57, 63], suggesting functional diversity that can be exploited for insect resistance.

Breeding efforts with *S*. *pennellii* accession LA716 led to the development of an acylsugar breeding line, CU071026, that accumulates moderate amounts of acylsucroses [64]. Evaluation of CU071026 and related germplasm with additional QTL affecting amount of acylsugars accumulated demonstrated improved resistance to whiteflies compared to non-acylsugar controls [54, 64]). Additional QTL from *S*. *pennellii* LA716 that impact acylsugar chemistry [61, 65–67] were introgressed to generate a set of mono-introgression addition lines in the CU071026 background that produce moderate-high amounts of acylsucrose acylsugars with distinct alterations in fatty acid profiles [68]. Further combinations of these fatty acid QTL in the CU071026 background generated additional acylsugar lines with more complex acylsugar fatty acid profiles [69].

It is assumed that moderating the abundance or changing the behavior of an insect that vectors plant viruses, like whiteflies, can consequently reduce virus incidence; however, no studies have been able to show that acylsugar mediated resistance in a cultivated tomato background significantly reduces whitefly incidence and or survival in a field setting. The closest report to a field trial was field cage studies [54] on the impact of different acylsugar amounts of a set of lines in the CU071026 background on oviposition by sweetpotato whitefly. The authors of two studies [70, 71] have shown that tomatoes containing insect resistance genes from *S*. *pimpinellifolium* that allow accumulation of acylsucroses exhibit a reduction in TYLCV compared to negative controls, but these studies were not performed in field settings. The authors of an additional laboratory-based study [72] utilizing CU071026 and related germplasm demonstrated the ability of acylsugars to reduce whitefly preference and fitness and subsequently reduce the ability of whiteflies to acquire and transmit TYLCV. The question remained whether these results would translate to a reduction in TYLCV incidence in the field.

The effectiveness of acylsugar-mediated resistance in the field could be impacted by environmental conditions because acylsugars are secreted from trichomes as naked droplets, which could be dislodged by heavy rainfall and high-pressure sprays, either or both of which can occur in numerous tomato production areas. In addition to acylsugar amounts, work is needed on the impact of acylsugar chemistry in planta on insects and insect transmitted viruses. A laboratory study [73] using purified acylsugars from CU071026 and several *S*. *pennellii* accessions on detached leaflets demonstrated that the chemistry of acylsugars, such as the sugar backbone or fatty acid profile, also had an impact on whitefly oviposition. However, impacts of sugar backbone or acylsugar fatty acid profile on whiteflies in the field have also not been documented. It is critical that the amount and fatty acid profile of acylsugars in field settings be evaluated not only for impact on reducing whitefly incidence and survival, but most importantly on reducing incidence of TYLCV to demonstrate the effectiveness of acylsugars in a typical tomato production environment.

We hypothesized that variation in the amount of acylsugars and alterations to the profile of the fatty acid components of the acylsugars secreted from glandular trichomes of tomato plants would influence whitefly oviposition behavior, nymph survival, and incidence of whitefly transmitted TYLCV in a field setting. To test this hypothesis, we conducted field trials with tomato germplasm containing introgressed QTL and with hybrids between cultivated tomato and *S*. *pennellii* accessions; these entries reliably alter the amount and fatty acid profile of secreted acylsugars. The purpose of this study was to test the utility of acylsugars as a method of white fly and TYLCV control in the field and whether increased acylsugar amount the presence of specific fatty acid profiles, or both could be important in mediating this defense/tolerance.

## Materials and methods

### Tomato entries used in experiments and growth conditions

The commercial tomato hybrid “Florida-47” (FL-47) and the entry FLX-TY3 (Fla. 7946 NIL heterozygous for Ty-3) were supplied by the University of Florida’s Gulf Coast Research and Education Center (GCREC) and have simple trichomes, only a few of which bear detectable droplets; these lines accumulate only trace amounts of acylsugars compared to the acylsugar-producing entries and were included as negative controls. Seeds of other tomato entries were produced at Cornell University [64]. The Cornell benchmark acylsugar-accumulating line CU071026 was used as an acylsugar control. Lines FA2/AS, FA7/AS and FA8/AS each possess the five standard introgressions of CU071026 and one additional introgression with QTL altering fatty acid profiles [68, 69]. FA2/FA8/AS and FA2/FA7-AS7/AS contain 5 and 4 of the CU071026 introgressions, respectively, and contain two additional introgressions that alter acylsugar fatty acid profiles [69].

Lines developed for increased trichome density, and/or acylsugar production have the prefix “AL” and include AL-sib, AL10/SW5/AS, AL10b/SW5/AS, AL10/AS, AL6/SW5/AS, AL6/AS, and AL6/AL10/AS; these lines are highly related to CU071026 [54]. Entries with the prefix “ISX” are hybrids between NC33EB1 and four *S*. *pennellii* accessions (ISX-1-4) or between CU071026 and three *S*. *pennellii* accessions (ISX-6-8). Seeds of the *S*. *pennellii* accessions were obtained from the TGRC center.

Two of the entries, ASX-1 and ASX-2 were acylsugar tomato hybrids, each of which was developed by crossing two different acylsugar producing tomato lines that differ for one or more introgression for increased trichome density or for altered acylsugar amount. Both hybrids, ASX-1 and ASX-2, share a female parent that has the same *S*. *pennellii* introgressions as CU071026 plus the chromosome 6 QTL (QTL6) that increases trichome density and acylsugar amount [54]. The differences in the two hybrids are due to their different male parents. The male parent of ASX-1 has an additional introgression intended to introduce the TSWV resistance gene, Sw-5, to the hybrid; the addition of this introgression to the line did not alter acylsugar amount. The male parent of the hybrid ASX-2 possesses a large (>20Mb) introgression carrying the Tm-2 gene, for resistance to *Tomato mosaic virus*; serendipitously, transfer of this introgression to the acylsugar line CU071026 was associated with significantly increased acylsugar amount. This is not to imply that the Tm-2 gene is responsible for the increased acylsugar level, but rather that one of many genes in this >20Mb introgression resulted in increased acylsugar level. In addition, the male parent for hybrid ASX-2 also has a modification in its chromosome 3 *S*. *pennellii* introgression, replacing the *S*. *pennellii* DNA in the upper 2 Mb of the introgression with tomato DNA; this change is associated with ca 10–15% reduction in total acylsugar amount when present in CU071026 background. Therefore, the male parent of hybrid ASX-2 had two features that would raise and lower acylsugar amount, respectively. Hybrid ASX-3 is a hybrid between two acylsugar lines, one of which has the same modification in its chromosome 3 *S*. *pennellii* introgression, which lowers the amount of acylsugars accumulated but does not possess the Tm-2 containing introgression. Hybrid ASX-3 is also heterozygous for Ty-3, which was carried by the male parent. Entry TY3/TM2/AS is a homozygous acylsugar line containing the standard 5 CU071026 introgressions and is also homozygous for the Ty-3 and Tm-2 containing introgressions. A list of all entries included in the field trials is included in S1 Table.

Seeds were sown in Speedling (Ruskin, FL, USA) potting mix in 128-cell trays and maintained for 5–6 weeks in a greenhouse, where they were treated with Plantex (Brampton, ON, Canada) 15-15-18 fertilizer every two weeks. Seedlings were transplanted into the field on 15 Apr for the spring 2014 trial, 22 Sept for the fall 2014 trial, and 11 Mar for the spring 2015 trial. Seedlings were transplanted into 1.5m wide beds covered in white plastic mulch, spaced 46 cm apart. The soil type is Myakka fine sand. Plants were staked and tied and irrigated using drip tape underneath the plastic mulch. Insecticides were not applied to plants; fungicides were applied as needed to manage foliar diseases.

### Experimental design

Field trials were carried out in the spring and fall of 2014 and the spring of 2015 at the GCREC in Balm, Florida, to evaluate the relationship between tomato entry, acylsugar composition and acylsugar amounts on whitefly oviposition, nymph survival/development, and incidence of TYLCV. Fourteen entries were evaluated in spring 2014 and fall 2014, and 11 in spring 2015. Each entry was considered a treatment and was planted in a row of fourteen plants. Each treatment was replicated four times and arranged in a randomized complete block design. Some entries were repeated across experiments for reference, but the set of entries was altered each season to incorporate new germplasm for evaluation. The TYLCV-susceptible commercial tomato variety Florida-47 was used as a negative control in each trial, and FLX-TY3 was used in each trial in 2014. Whiteflies were naturally present and not managed or introduced into the field trials.

### Whitefly egg and nymph abundance

Early, mid- and late season leaflet samples were collected to assess treatment impacts on densities of whitefly eggs and nymphs. Leaflet samples were collected 5 and 27 May and 9 June in spring 2014, corresponding to days after planting (DAP) of 20, 42 and 55 days, respectively; 15 Oct, 4 and 24 Nov in fall 2014, corresponding to DAP of 23, 43 and 63 days, respectively; and 13 and 27 Apr and 18 May in spring 2015, corresponding to DAP of 33, 47 and 68 days, respectively. One leaflet was collected from ten consecutive plants of each entry in each plot per sample date. Samples were collected from the third fully expanded leaflet from the top of the plant for the first sample. The 7^th^ or 8^th^ leaflet from the top of the plant was selected for mid and late season samples. Leaflets were examined using a stereomicroscope in the laboratory and the number of whitefly eggs and nymphs per leaflet was recorded.

### Tomato yellow leaf curl virus assessment

Visual virus assessments were made twice for the spring 2014 trial in weeks 5 and 7 after planting and samples for PCR evaluation were collected 5 June (51 DAP). Visual virus assessments were made six times for the fall 2014 trial in weeks 3,5,6,7,8,9 after planting and samples for PCR evaluation were collected 12 November (51 DAP). Visual virus assessments were made 4 times for the spring 2015 trial in weeks 4,5,6,7 after planting and samples for PCR evaluation were collected 1 May (51 DAP). TYLCV presence was assessed on a subsample of leaflets (between 20–50% of plants tested) via PCR using primers designed from a highly conserved region of the TYLCV coat protein gene and verified through sequencing as detailed in [74]. Visual assessment of TYLCV presence supported the PCR results: plants were considered TYLCV infected when they exhibited clear symptoms of the virus: bright yellow leaflet margins, stunted, bright yellow leaflets, and shorted petioles.

### Acylsugar amounts and fatty acid profile

Amounts of accumulated acylsugars for all entries were measured on field grown 7–12 week old plants similar to the method discussed in Leckie et al. [54, 67]. A total of 10 samples of two primary lateral tomato leaflets were collected from each of the four reps in each field trial, totaling 40 samples per entry per experiment. Leaflets were re-dried after rinsing and then weighed, so that acylsugar amount could be expressed per weight dried leaf tissue. The amount of acylsucrose and acylglucose from each sample was ascertained and expressed as absolute amount and or as a percentage of the acylsugar amount of the benchmark acylsugar line [CU071026]. Measured acylsugar accumulation from each entry for each season was utilized to assign each entry to one of four categories ranging from “0” to “3”, with “0” indicating the standard tomato entry(s) with trace acylsugar accumulation, “1” indicating low, “2” indicating moderate and “3” indicating high acylsugar accumulation. These groupings were used for further analyses to investigate the role of acylsugar amount in whitefly response and TYLCV incidence.

Variation for the acylsugar fatty acid profile of each entry was ascertained by the methods detailed in [67–69].

### Statistics

Hierarchical clustering data analysis of the acylsugar fatty acid data from each tomato entry was conducted using Pearson correlation using pairwise average-linkage clustering for entries using the tools provided by GenePattern [75]. This analysis was conducted to group the tomato entries based on the composition of acylsugar fatty acids and shows the relationship between entries based on the relative proportion of each individual fatty acid’s accumulation when compared to other entries, rather than comparing the total amount of fatty acid or the percentage of the fatty acid accumulating within an entry. It also provides information about which acylsugar fatty acids are more variable or more consistent across entries. Data from the same entries across experiments was pooled.

The average abundance of whitefly eggs and nymphs across the timepoints of each experiment for entries grouped by amount of acylsugar accumulation was analyzed using a linear model fit in R using the acylsugar amount grouping of entries as the independent variable. Least square means estimates were generated using the lsmeans package in R [76] and means compared using Tukey’s HSD. The abundance of whitefly eggs and nymphs were analyzed as log-normally distributed variables.

The average abundance of whitefly eggs and nymphs across all entries was analyzed using a linear model in R with entry and experiment as independent variables. Least square means were estimated using lsmeans in R and Tukey’s HSD was used to compare entries. The abundances of whitefly eggs and nymphs were analyzed as log-normally distributed variables.

Likelihood of infection by TYLCV across entries present in each of the three experiments were analyzed using a general linear model (logit with binomial distribution) with entry and experiment as the independent variables. Each plant was treated as a bernoulli trial where a plant was determined to be positive or negative for the virus via PCR. Least square mean estimates of the probability of a plant of a given entry being positive for TYLCV were estimated, and Tukey’s HSD was used to compare entries. The TYLCV infection frequencies were converted to log-odds and analyzed as log-normally distributed variables before estimating probability of TYLCV infection.

To study the role of the acylsugar fatty acid profile in affecting abundance of whitefly eggs and nymphs, as well as the proportion of plants infected with TYLCV, variable selection using minimum-AIC (Akaike Information Criterion) was conducted leading to models fit using the independent variables detailed below. For abundance of whitefly eggs and nymphs, linear models were fit in R, and eggs and nymphs were analyzed as log normally distributed variables. For incidence of TYLCV, a general linear model (logit) with binomial distribution was run in R. For abundance of whitefly eggs, variable selection led to a model fit using the independent variables presented in S2 Table. For abundance of whitefly nymphs, variable selection led to a model fit using the independent variables presented in S3 Table. For incidence of TYLCV, variable selection led to a model fit using the independent variables presented in S4 Table. For selection of the best fit model for each resistance metric we utilized a cutoff of α <0.01 when comparing models using loglikelihood to select the most accurate models that minimized AIC. Variables for all three models were selected from the following pool: experiment, total acylsugar amount (entry wise estimate g^-1^ leaf weight), acylglucose amount, averages of acylsugar fatty acid amounts (Umol g^-1^ leaf weight) across entries for each of the measured fatty acids, averages of the total amount of all acylsugar fatty acids in Umol g^-1^ leaf weight for each entry, and averages of the relative percent of each acylsugar fatty acid out of the total amount of fatty acids of an entries’ fatty acid profile across entries. Only fatty acids that constituted at least one percent of the total acylsugar fatty acids of at least one entry’s profile were included in the variable selection and modeling. Only variables selected by minimum-AIC with a significance of p < 0.01 were analyzed as independent variables in analysis of covariance (ANCOVA) for whitefly eggs and nymphs and analysis of deviance for incidence of TYLCV. Amount of acylsugar was included as a variable in the ANCOVA to demonstrate impact of the minimum-AIC selected variables after accounting for amount of acylsugar.

GGplot in R [77] was utilized to create Figs 2–6.

## Results

### Acylsugar amounts across entries and field trials

Variation was observed across entries within each season and between field trials for the amounts of acylsugars accumulated; the range of values were about an order of magnitude between the highest and lowest acylsugar-accumulating entries (Table 1). Differences between entries was expected; variation within an entry for amounts of acylsugars across seasons was also observed and expected due to the impact of the environment on accumulation. Variation of temperature and light intensity would impact accumulation of acylsugars; driving rainfall and wind-blown particles could also have some impact. These factors are expected to vary from day to day within and across seasons. Spring seasons transition from cooler to warmer temperatures and fall seasons are the opposite. This variation was accounted for in analyses.

**Table 1:** The average amounts of total acylsugars (AS) (± SEM) accumulated by each entry for each field trial season in umol g^-1^ weight leaf tissue. Measured acylsugar accumulation from each entry for each season was utilized to assign each entry to one of four categories ranging from “0” to “3”, with “0” indicating the standard tomato entry(s) with trace acylsugar accumulation, “1” indicating low, “2” indicating moderate and “3” indicating high acylsugar amount accumulation. Within each season, entries not connected by the same letter are significantly different (a = 0.05) Tukey HSD.

Spring 2014			Fall 2014			Spring 2015		
Entries	Amount AS Accumulated	AS Amount Grouping	Entries	Amount AS Accumulated	AS Amount Grouping	Entries	Amount AS Accumulated	AS Amount Grouping
ISX-7	12.2 ± 1.1 a	3	ISX-6	31.5 ± 1.1 a	3	AL6/AL10/AS	22.5 ± 0.8 a	3
ISX-6	11.3 ± 1.0 a	3	ISX-7	26.8 ± 1.5 ab	3	AL6/AS	18.5 ± 0.9 ab	3
ASX-1	10.5 ± 0.7 a	3	ISX-8	22.6 ± 1.1 b	3	FA7/AS	16.3 ± 1.2 ab	3
ASX-2	8.1 ± 0.6 ab	2	AL6/SW5/AS	11.2 ± 1.1 c	3	AL10/AS	14.0 ± 1.2 bc	3
ISX-8	8.1 ± 0.6 ab	2	ASX-1	6.9 ± 0.6 d	2	AL-sib	9.9 ± 0.9 d	2
CU071026	5.7 ± 0.6 bc	2	FA7/AS	6.5 ± 0.4 d	2	FA2/FA7-AS7/AS	9.4 ± 0.8 cd	2
TY3/TM2/AS	4.1 ± 0.4 cd	1	CU071026	6.3 ± 0.7 de	2	CU071026	7.7 ± 0.6 de	2
ISX-3	3.9 ± 0.5 cd	1	TY3/TM2/AS	5.5 ± 0.5 d-f	2	FA2/AS	6.2 ± 0.7 ef	2
FA2/AS	3.1 ± 0.4 de	1	AL10b/SW5/AS	5.2 ± 0.5 d-f	2	FA8/AS	5.8 ± 0.5 ef	2
ASX-3	2.9 ± 0.3 de	1	ASX-2	4.5 ± 0.4 d-f	2	ASX-3	4.4 ± 0.5 f	1
ISX-1	2.4 ± 0.3 de	1	AL10/SW5/AS	4.3 ± 0.5 ef	1	FA2/FA8/AS	2.8 ± 0.3 g	1
FA8/AS	2.4 ± 0.4 ef	1	FA2/AS	3.5 ± 0.3 fg	1	FL-47	0.7 ± 0.0 h	0
ISX-2	2.0 ± 0.2 e	1	FA8/AS	2.5 ± 0.3 g	1			
ISX-4	1.7 ± 0.2 e	1	ASX-3	2.2 ± 0.3 g	1			
FLX-TY3	0.5 ± 0.2 fg	0	FL-47	0.9 ± 0.2 h	0			
FL-47	0.2 ± 0.1 g	0	FLX-TY3	0.6 ± 0.1 h	0			

As expected, the standard tomato entries (FLX-TY3 and FL-47) accumulated only trace amounts of acylsugars as is common for standard tomato lines. For the Spring 2014 experiment the effect of entry on acylsugar accumulation was highly significant (Type I Sum of Squares = 9098.7, p < 0.0001). For the fall 2014 and spring 2015 trials, the effect of entry on acylsugar accumulation was again significant (Type I Sum of Squares = 53462; p < 0.0001, Type I Sum of Squares = 18847, p < 0.0001, respectively. Comparison across the entries for each experiment revealed differential accumulation of the amount of acylsugars; entries repeated in more than one experiment had a similar pattern of acylsugar accumulation (Table 1). Some of the interspecific entries (ISX-6 and ISX-7) and entries (ASX-1, AL6/AS, AL6/SW5/AS, AL10/AS, and AL6/AL10/AS) that contained QTLs known to increase acylsugar amounts [54] had higher acylsugar accumulation. The acylsugar amounts for entries within each experiment were used to assign the entries to one of four acylsugar amount groupings (described in methods).

#### Acylsugar chemotypes across entries and field trials

**Fig 1:**
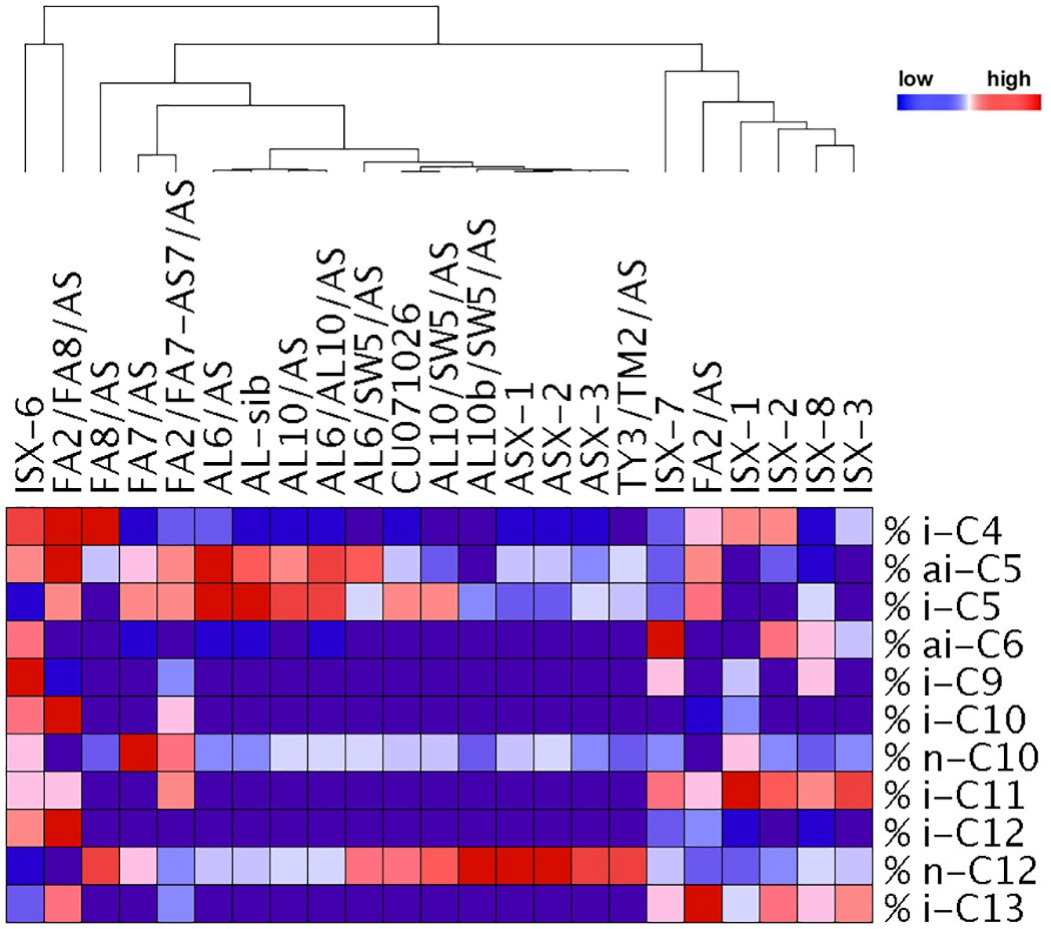
Hierarchical clustering of included tomato entries by relative abundance of acylsugar fatty acids, where each fatty acid constitutes a percentage of the total fatty acid profile for a given entry. Fatty acids included make up at least 1 percent of a given entries’ total fatty acid profile. Pearson correlation used to separate entries. Within a row, colors are standardized across columns, allowing comparisons of the relative proportion of an individual fatty acid across entries, but does not reveal the absolute amount of the acylsugar fatty acids (see S3 Fig for this information). Comparisons within a column are not relevant, because the colors do not convey relative proportion of each fatty acid within an entry (see S2 Fig for this information). Color represents the relative abundance of an acylsugar fatty acid for a given entry with red indicating higher accumulation compared to the other entries in the row, and blue indicating a lower relative percent accumulation of a fatty acid compared to the other entries. Purple represents fatty acids that could not be detected for a given entry. Entries with the prefix "FA" have modified fatty acid profiles and entries with the prefix "ISX" are interspecific hybrids with modified fatty acid profiles; these two groups displayed the most unique fatty acid profiles and clustering patterns.

The entries in each experiment also displayed a diverse array of acylsugar chemotypes. Differences between entries was expected as entries were selected for inclusion based on known and consistent variation for acylsugar chemotype. A chemotype refers to the acylsugar backbone (acylsucrose, acylglucose or a mixture) and the array of fatty acids esterified to the acylsugar backbones (fatty acid profile). The interspecific entries in the spring and fall trials of 2014 accumulated acylglucoses in addition to acylsucroses, whereas all other entries accumulated solely acylsucroses (S1 Fig). Analysis of the relative abundance (each acylsugar fatty acid as a percent of the total profile) of acylsugar fatty acids for entries across experiments revealed clustering patterns and demonstrated the consistency of QTLs impacting fatty acid profile across experiments (Fig 1). Specifically, the entries separated into three main groups, with entries ISX-6 and FA2/FA8/AS in one group exhibiting the most unique fatty acid profiles. The second group consisted of the interspecific entries ISX-1, ISX-2, ISX-3, ISX-7, ISX-8 and the acylsugar tomato line, FA2/AS; the third group consisted of all other entries, such as CU071026 and many closely related entries. Within the largest group of entries more closely related to CU071026, sub clusters revealed FA8/AS possessed a distinct fatty acid profile, as did the cluster of the entries FA7/AS and FA2/FA7-AS7/AS. The remaining entries, very closely related to CU071026, did not show any clear separation in the cluster analysis, and displayed similar profiles. Percent bar plots of the acylsugar fatty acid profiles of all entries, support the hierarchical clustering analysis (HCA) data and revealed that the acylsugar profiles of most entries were dominated by the presence of ai-C5, i-C5 and n-C12 fatty acids (S2 Fig). CU071026 and many related entries (ASX-1, ASX-2, ASX-3, AL6/AS, AL6/AL10/AS, AL10/AS, AL10b/AS, AL-sib, and TY3/TM2/AS) had profiles that were quite similar and almost exclusively consisted of these three acylsugar fatty acids. In contrast, many of the interspecific entries and some breeding lines accumulated acylsugars with altered proportions of novel fatty acids not typically seen in the acylsugars of the benchmark entry, CU071026, and related entries. An additional analysis revealed the absolute abundance of predominant acylsugar fatty acids among the entries each season (S3 Fig) and demonstrated that several interspecific entries, such as ISX-6, ISX-7 and ISX-8, as well as several acylsugar lines and hybrids (ASX-1, ASX-2, AL6/SW5/AS, AL6/AS, AL6/AL10/AS) accumulated much greater amounts of particular acylsugar fatty acids, as well as a greater total amount. Entry ISX-4 and the standard tomatoes FL-47 and FLX-TY3 did not produce sufficient acylsugars to allow reliable evaluation of fatty acid profile.

### Whitefly egg and nymph abundance across entries and experiments

There were differences in total numbers of whitefly eggs and nymphs between seasons; in the highest season there were approximately 10x more whitefly eggs and nymphs compared to the season with the lowest pressure; the observed variation was expected due to natural infestation. Analysis of variance for the impact of entry and experiment demonstrated that each factor explained a significant amount of the observed variation for the abundance of whitefly eggs (Type III Sum of Squares = 4,901,019 and 807,888, respectively, p<0.0001). Considerable differences were observed among the entries for abundance of whitefly eggs. In an analysis combining all entries across the three experiments, it was observed that several of the acylsugar-accumulating entries displayed fewer whitefly eggs compared to the standard tomato controls (FL-47 and FLX-1) (Table 2). Numerous entries containing the AL6 QTL [54] (AL6/SW5/AS, ASX-1, AL6/AS, AL6/AL10/AS, and ASX-2) were among the entries with the lowest abundance of whitefly eggs; the FA2/FA7-AS7/AS, ISX-6 and ISX-7 entries also displayed low whitefly egg abundance. Several of the interspecific entries (ISX-1, ISX-2, ISX-3 and ISX-4) displayed an increased abundance of whitefly eggs, particularly ISX-4.

**Table 2:** Least square mean estimates of the number of whitefly eggs (± SEM) from 10 leaflet counts averaged across field trials/seasons. Abundance of eggs was analyzed as a log-normally distributed variable. Entries not connected by the same letter are significantly different (a = 0.05) Tukey HSD.

Entries	Number of whitefly eggs
AL6/SW5/AS	3.83 ± 1.49 a
FA2/FA7-AS7/AS	4.67 ± 1.86 a-c
ASX-1	5.26 ± 1.35 a
ISX-6	5.63 ± 1.44 a
AL6/AS	5.72 ± 2.28 a-d
AL6/AL10/AS	7.05 ± 2.81 a-e
ISX-7	7.12 ± 1.82 ab
ASX-2	7.33 ± 1.87 ab
TY3/TM2/AS	10.41 ± 2.66 a-d
FA7/AS	11.34 ± 3.08 a-e
AL10b/SW5/AS	11.55 ± 4.50 a-f
AL10/AS	11.61 ± 4.63 a-f
FA2/FA8/AS	12.11 ± 4.83 a-f
CU071026	13.54 ± 2.81 a-e
FA2/AS	15.74 ± 3.26 a-f
ASX-3	16.61 ± 3.49 a-f
ISX-8	18.51 ± 4.73 a-f
AL-sib	21.40 ± 8.53 a-g
FLX-1	27.69 ± 7.08 c-f
FA8/AS	27.69 ± 5.74 d-f
FL-47	30.26 ± 6.28 d-f
AL10/SW5/AS	34.24 ± 13.36 b-g
ISX-3	37.95 ± 12.95 d-g
ISX-1	50.87 ± 17.36 e-g
ISX-2	63.48 ± 21.66 fg
ISX-4	131.32 ± 44.80 g

Analysis of variance for the impact of entry and experiment demonstrated that each factor explained a significant amount of the observed variation for the abundance of whitefly nymphs (Type III Sum of Squares = 3,129,652 and 577,502, respectively, p<0.0001). Considerable differences were observed among entries for the abundance of whitefly nymphs and an analysis combining entries across experiments demonstrated that fewer nymphs were observed on numerous entries compared to the standard tomato controls (Table 3). Entries containing the AL6 QTL (ASX-1, AL6/AL10/AS, AL6/SW5/AS, AL6/AS and ASX-2) were among the entries with the lowest abundance of nymphs; AL10b/SW5/AS also displayed a low abundance of nymphs. Two interspecific entries (ISX-6 and ISX-7) also displayed lower whitefly nymph abundance than the standard tomato controls and several of the other interspecific entries.

**Table 3:** Least square mean estimates of the number of whitefly nymphs (± SEM) from 10 leaflet counts averaged across field trials/seasons. Abundance of eggs was analyzed as a log-normally distributed variable. Entries not connected by the same letter are significantly different (a = 0.05) Tukey HSD.

Entries	Number of nymphs
ASX-1	3.10 ± 0.90 a
AL6/AL10/AS	3.85 ± 1.75 a-d
AL6/SW5/AS	3.95 ± 1.76 a-c
AL6/AS	4.16 ± 1.89 a-d
ISX-7	5.10 ± 1.49 ab
AL10b/SW5/AS	5.80 ± 2.58 a-d
ISX-6	5.89 ± 1.72 a-c
ASX-2	6.56 ± 1.91 a-d
TY3/TM2/AS	7.85 ± 2.29 a-d
ISX-8	8.19 ± 2.39 a-d
FA7/AS	8.40 ± 2.60 a-d
FA2/FA7-AS7/AS	8.76 ± 3.98 a-g
CU071026	8.98 ± 2.12 a-d
AL-sib	11.29 ± 5.13 a-g
AL10/AS	11.51 ± 5.23 a-g
AL10/SW5/AS	11.75 ± 5.22 a-g
FA2/AS	11.79 ± 2.79 a-e
ASX-3	13.87 ± 3.28 b-f
FA2/FA8/AS	15.47 ± 7.03 a-g
FA8/AS	21.08 ± 4.98 c-g
ISX-3	21.87 ± 8.50 b-g
ISX-1	37.10 ± 14.42 d-g
FLX-1	44.91 ± 13.09 e-g
FL-47	47.82 ± 11.30 g
ISX-2	58.96 ± 22.92 e-g
ISX-4	69.52 ± 27.03 fg

### The effect of acylsugar amount and composition on whitefly egg abundance

Analysis using the acylsugar amount groupings (Table 1) indicated a negative relationship between acylsugar amount and whitefly egg abundance and helped to explain the egg abundance variation among entries across the count dates. The Spring 2014 experiment, in which the commercial tomato controls averaged more than 400 eggs per 10 leaflets, revealed a general pattern of increasing whitefly egg counts over the course of the experiment (S4 Fig); however, the egg counts for the tomato controls dropped precipitously between the 2^nd^ and 3^rd^ count. The egg count/10 leaflet values for the entries with either moderate or high amounts of acylsugars increased slowly over time, and were significantly lower for the second count, but not the third due to the severe decline in numbers on the susceptible control, The entries with only low acylsugar amounts exhibited steadily increasing egg abundance over time, with mean egg count/10 leaflet values that were consistently higher than the moderate and high acylsugar-accumulating entries for each collection date.

The Fall 2014 experiment, which had the lowest insect pressure, revealed that the high acylsugar-accumulating entries did not separate well from the other groupings until the final collection date when the high acylsugar-accumulating entries displayed lower whitefly egg abundance compared to the standard tomato and low acylsugar-accumulating entries (S5 Fig). The Spring 2015 experiment had lower whitefly oviposition pressure and all acylsugar amount groupings displayed similar whitefly egg abundance for each collection date (S6 Fig).

Variation in abundance of whitefly eggs across all experiments for the acylsugar-accumulating entries can be best described by three variables chosen through minimum-AIC: experiment, the relative proportion of one acylsugar fatty acid, ai-C5, and the amount of one acylsugar fatty acid, n-C12 (S2 Table). Experiment explained the most variation (Type III Sum of Squares = 105.57, p<0.001). The relative proportion of ai-C5, and the amount of n-C12 fatty acids were found to explain a significant amount of additional variation, after accounting for experiment and amount of acylsugar (Type III sum of squares: 19.36, p = 0.002 and 64.44, p <0.001, respectively). Amount of acylsugar was not significant when included in this model (Type III sum of squares = 1.57, p = 0.368). Partial regression coefficients indicated that both percent ai-C5 and amount of n-C12 fatty acids were negatively correlated with abundance of whitefly eggs (t = -3.168 and -5.781, respectively).

### The effect of acylsugar amount and composition on whitefly nymph abundance

**Fig 2:**
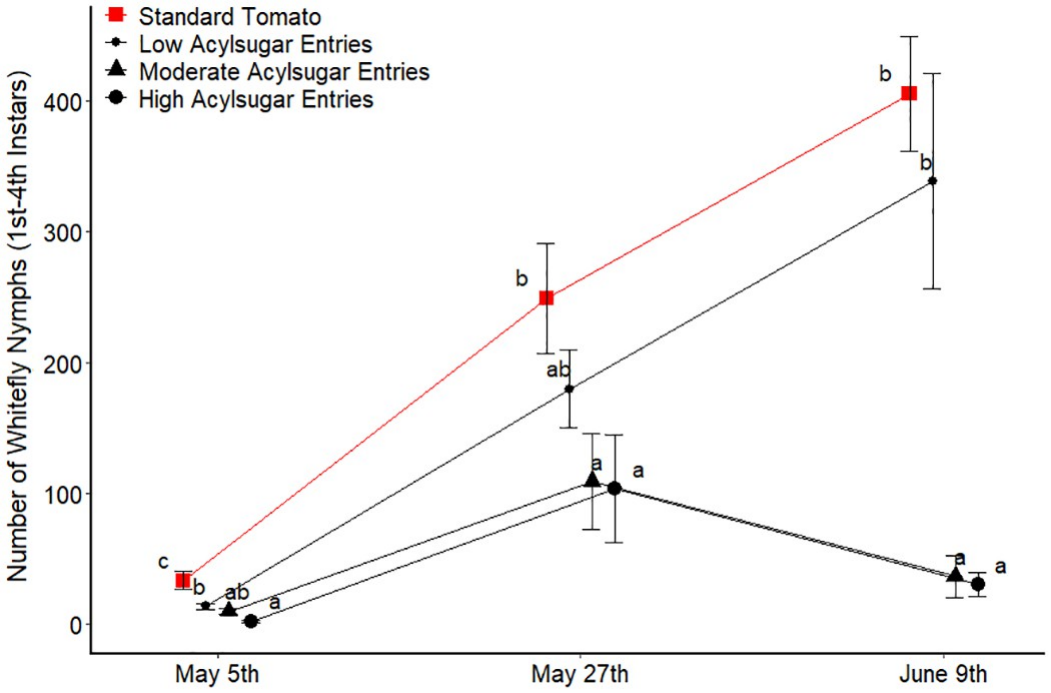
Number of whitefly nymphs in Spring 2014 trial for each acylsugar amount grouping of entries. Four replicates pooled for each entry at each count date and averaged across entries within each acylsugar amount grouping at each count date. For each count date, acylsugar amount groupings of entries not connected by the same letter are significantly different (a = 0.05). Error bars represent one standard error of the mean.

Analysis using the acylsugar amount groupings (Table 1) helped to explain the variation in whitefly nymph abundance among entries across the count dates and demonstrated a negative relationship between acylsugar amount and whitefly nymph abundance. Specifically, for the spring 2014 trial, we observed that the standard tomato entries had a greater abundance of whitefly nymphs compared to the other entry groupings at the first count date (Fig 2). The entries with the highest acylsugar accumulation were observed to support fewer nymphs compared to the low acylsugar entry group as well. A similar trend was observed for the second and third count date; in fact, a decrease in whitefly nymphs was observed in the moderate and high acylsugar entry groups from the second to third count dates, whereas the abundance of nymphs in the standard tomato entries and low acylsugar entries continued to increase from the second count date to the third.

**Fig 3:**
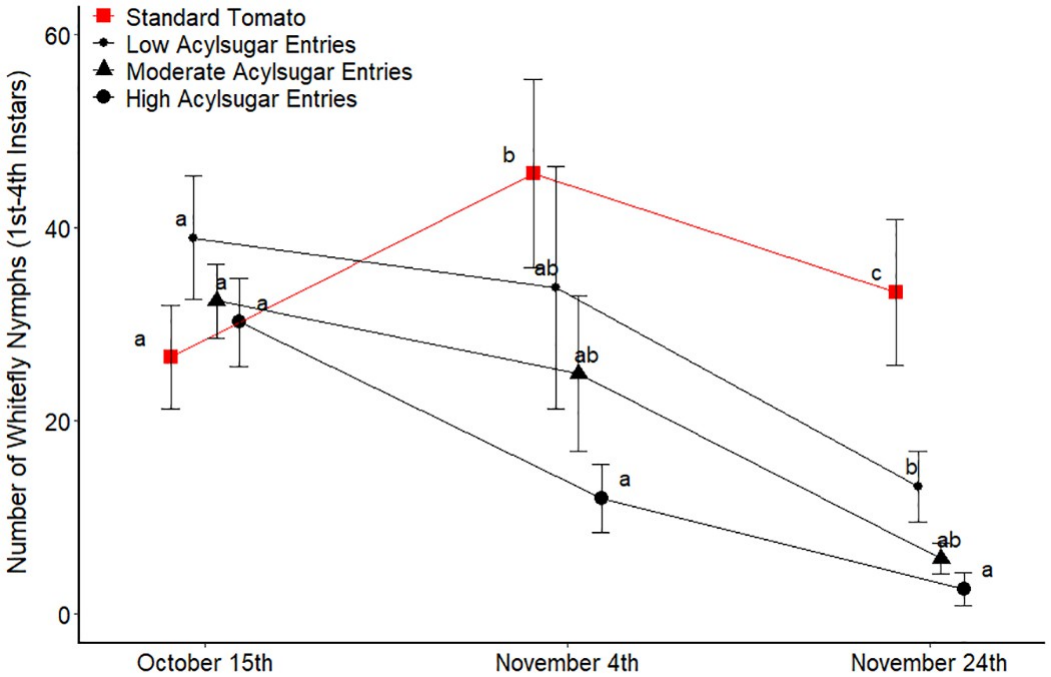
Number of whitefly nymphs per ten leaflets in Fall 2014 trial for each acylsugar amount grouping of entries. Four replicates from 10 leaflet samples pooled for each entry at each count date and averaged across entries within each acylsugar amount grouping at each count date. For each count date, acylsugar amount groupings of entries not connected by the same letter are significantly different (a = 0.05). Error bars represent one standard error of the mean.

The fall 2014 experiment revealed similar trends (Fig 3). For the first count date all entries displayed a similar abundance of whitefly nymphs; however, the second count date revealed a trend of decreasing whitefly nymph abundance as the amounts of acylsugars increased. For the second count date, the only statistical difference was between the standard tomato grouping and the high acylsugar group of entries. The third count date displayed the same trend with a greater abundance of whitefly nymphs on the standard tomato group compared to the other groups. Within the low, moderate and high acylsugar groupings, the high acylsugar entry grouping displayed fewer whitefly nymphs compared to the low acylsugar entry group, with the moderate acylsugar entry group intermediate to the low and high grouping.

**Fig 4:**
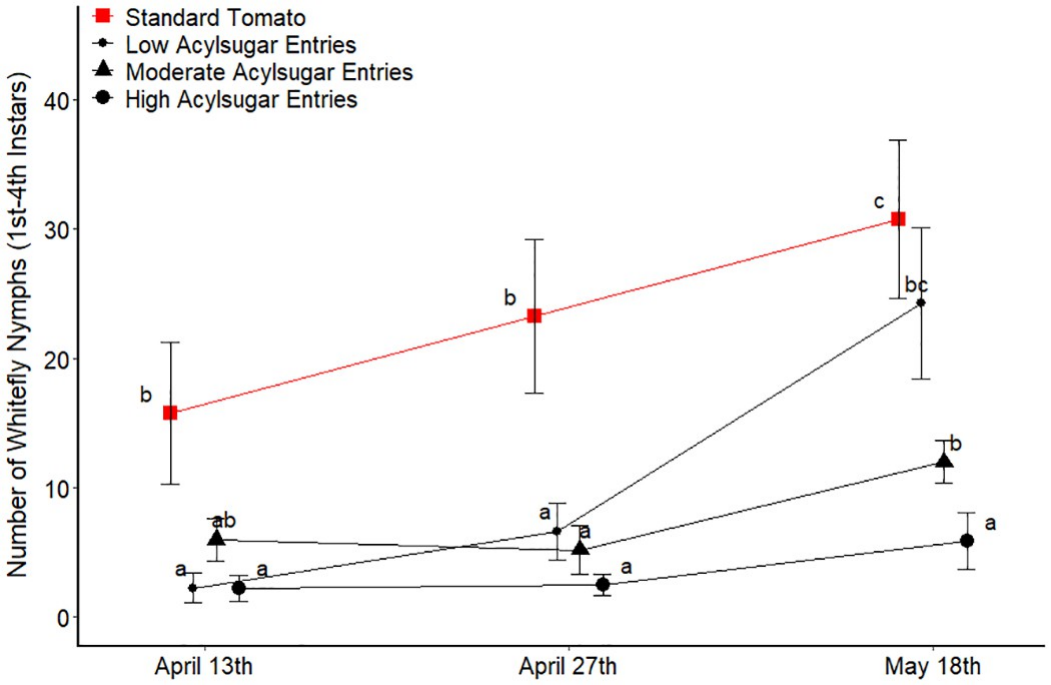
Number of whitefly nymphs in Spring 2015 trial for each acylsugar amount grouping of entries. Four replicates pooled for each entry at each count date and averaged across entries within each acylsugar amount grouping at each count date. For each count date, acylsugar amount groupings of entries not connected by the same letter are significantly different (a = 0.05). Error bars represent one standard error of the mean.

Analysis of the spring 2015 data revealed a pattern similar to the other seasons (Fig 4). For the first count date, relatively few whitefly nymphs were found on the low, moderate and high acylsugar entry groupings compared to the standard tomato entries. This same pattern held true for the second count date, with the standard tomato entries displaying higher whitefly abundance, whereas the other entries maintained a similar whitefly abundance as the first count. The third count revealed an increase in whitefly nymphs on the low acylsugar entry, which displayed a comparable abundance of nymphs as the standard tomato entries. In contrast, the moderate acylsugar entry grouping displayed fewer whitefly nymphs compared to the first two groups, with the high acylsugar entries displaying even fewer nymphs than the moderate acylsugar grouping.

Variation in abundance of whitefly nymphs across all experiments for the acylsugar-accumulating entries can be best described by two variables chosen through minimum-AIC: experiment and the amount of one acylsugar fatty acid, n-C12 (S3 Table). Experiment explained the most variation (Type III Sum of Squares = 208.45, p<0.001). The amount of n-C12 fatty acids were found to explain a significant amount of additional variation, after accounting for experiment and amount of acylsugar (Type III sum of squares: 69.23, p = 0.002). Amount of acylsugar was not significant when included in this model (Type III sum of squares = 1.66, p = 0.407). Partial regression coefficients indicated that the amount of acylsugar n-C12 fatty acids were negatively correlated with abundance of whitefly nymphs (t = -5.359).

### Variation for TYLCV incidence across entries and experiments

The entries in the spring 2014 trial were subjected to strong TYLCV pressure (as measured at 51 DAP) and most plants of the standard tomato controls, FL-47 and FLX-TY3, displayed symptoms of TYLCV. FLX-TY3 is heterozygous for the *Ty-3* TYLCV resistance gene; it appears this gene did not convey resistance in the heterozygous condition under heavy insect/virus pressure. In contrast, none of the TY3/TM2/AS entry plants tested positive for TYLCV; this acylsugar line is homozygous for the *Ty-3* resistance gene. None of the acylsugar lines, except TY3/TM2/AS, possessed complete resistance to TYLCV, however, considerable variation for TYLCV incidence was observed among the entries. Most of the acylsugar-accumulating tomato entries displayed plants positive for the virus, with entries CU071026 and ASX-3 having the lowest proportion of TYLCV positive plants. The interspecific entries appeared to have fewer virus positive plants, most notably the ISX-4 and ISX-7 entries had the lowest incidence of TYLCV.

The fall 2014 trial experienced less TYLCV pressure (as measured at 51 DAP), and most entries had a low proportion of TYLCV positive plants. FL-47 and FLX-TY3 had the highest incidence of TYLCV, while TY3/TM2/AS again had no plants test positive for TYLCV, as did the FA7/AS and ASX-1 entries. Across the interspecific entries, ISX-6 and ISX-7 displayed low incidence of TYLCV. The spring 2015 trial again experienced less TYLCV pressure (as measured at 51 DAP), and FL-47 had the highest incidence of TYLCV across all entries. All other entries displayed low TYLCV incidence, and several acylsugar-accumulating entries displayed no TYLCV incidence. The entries with no incidence of TYLCV included FA7/AS, FA8/AS, FA2/FA8/AS, FA2/FA7-AS7/AS, AL-sib, and ASX-3.

**Fig 5:**
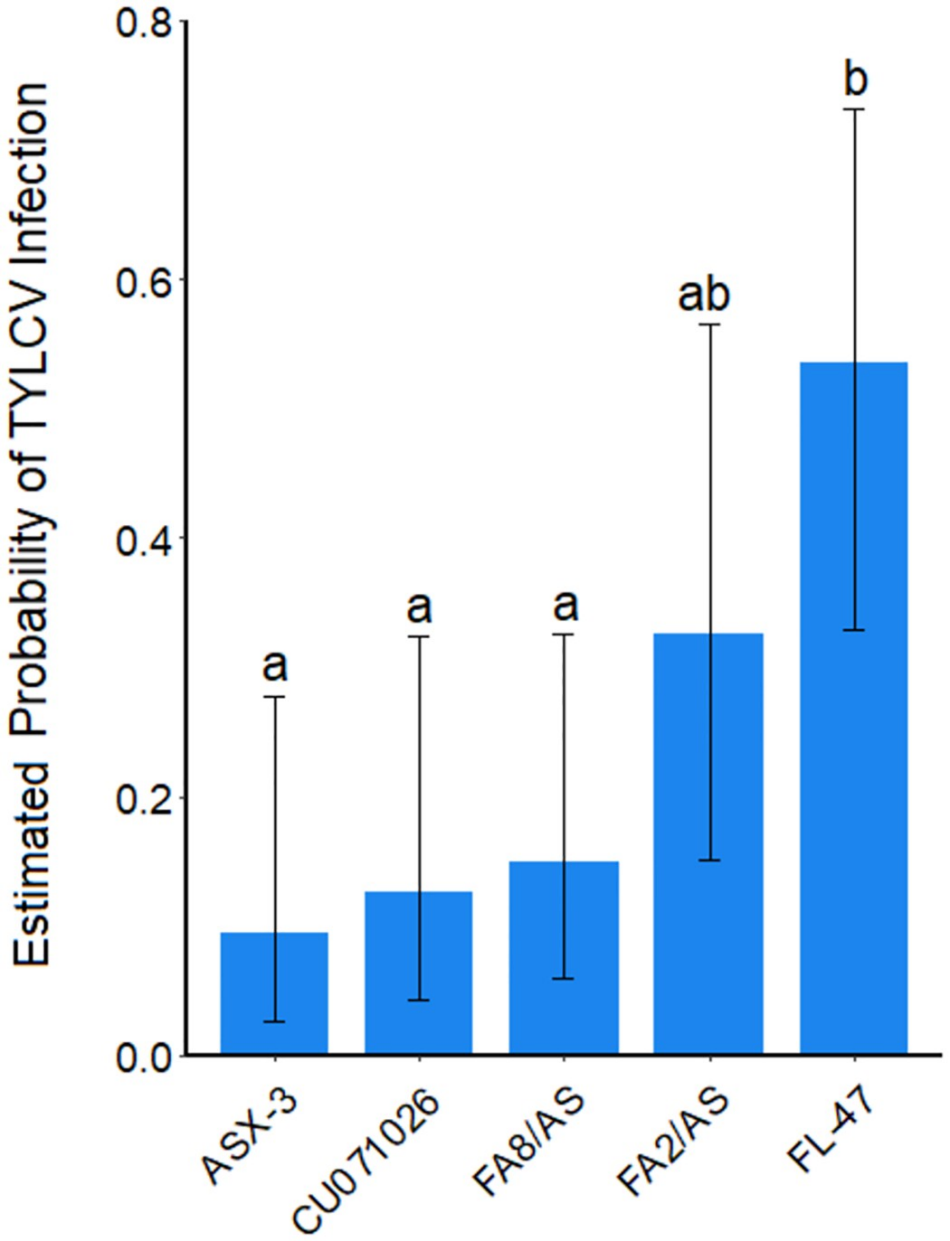
Estimated probability of tomato yellow leaf curl virus infection for 5 entries replicated across experiments at 51 days after planting. Estimated probabilities of virus infection are derived from the log odds, which are comparable to the observed incidence of virus infection. Entries not connected by the same letter represent significant differences (a = 0.05) for probability of virus infection (Tukey-adjusted comparisons). Log odds were utilized to generate LS means for statistical separation. Error bars represent 95% confidence intervals of the estimated LS means.

Five entries were repeated across all three experiments and incidence of TYLCV at 51 DAP for each entry was pooled across experiments and compared (Fig 5). The standard tomato, FL-47, exhibited the highest incidence of TYLCV; FA2/AS displayed a slightly lower but statistically similar incidence of TYLCV as FL-47 and was inseparable from the other entries. Conversely, the other three acylsugar entries: ASX-3, CU071026 and FA8/AS displayed a significantly lower incidence of TYLCV compared to FL-47.

### Association of acylsugar amounts and composition with TYLCV incidence

**Fig 6:**
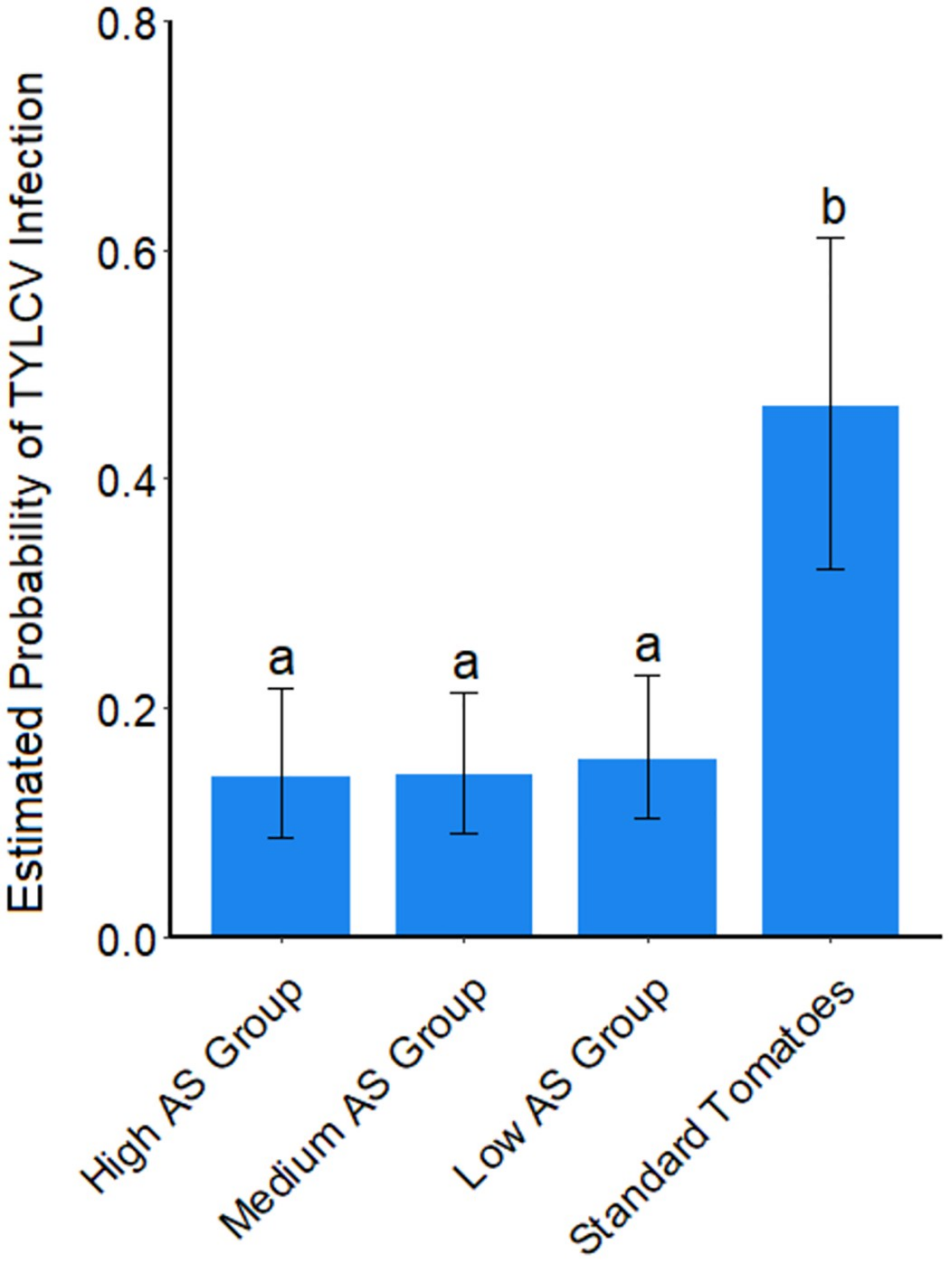
Estimated probability of tomato yellow leaf curl virus infection on groupings of field grown tomato plants based on amount of acylsugars accumulated at 51 days after planting. Estimated probabilities of virus infection are derived from the log odds, which are comparable to the observed incidence of virus infection. Groups not connected by the same letter represent significant differences (a = 0.05) for probability of virus infection across acylsugar amount groupings of entries (Tukey-adjusted comparisons). Log odds were utilized to generate LS means for statistical separation. Error bars represent 95% confidence intervals of the estimated LS means.

We investigated the association between varying acylsugar amounts and TYLCV incidence through an analysis of all entries across experiments, pooled by the acylsugar amount groupings in Table 1. Notably, the high, medium and low acylsugar amount groups all displayed a similar and lower frequency of TYLCV infection (as measured at 51 DAP) compared to the standard tomatoes (Fig 6).

Variation in incidence of TYLCV at 51 DAP across all experiments for the acylsugar-accumulating entries can be best described by a couple variables chosen through minimum-AIC: experiment, and the relative proportion of one acylsugar fatty acid, n-C10 (S4 Table). Experiment explained the most variation (likelihood χ^2^ = 106.739, p value <0.001), while acylsugar amount did not explain additional variation (likelihood χ^2^ = 0.868, p value 0.352), but was included to demonstrate the impact of the additional chosen variable (n-C10). The relative proportion of n-C10 was found to explain a significant amount of additional variation, after accounting for experiment and amount of acylsugar (likelihood χ^2^ = 11.452 p value < 0.001). Partial regression coefficients for the best fit model indicated that the relative proportion of n-C10 acylsugar fatty acids were significantly associated with a decrease in TYLCV incidence (estimate = -0.455). Conversely, among entries accumulating significant amounts of acylsugars, an increase in acylsugar amount was significantly associated with a slight increase in TYLCV incidence (estimate = 0.017).

## Discussion

While several studies have evaluated acylsugar-producing tomatoes or wild relatives of tomato and their control of whiteflies in laboratory or greenhouse settings, our study is the first to provide evidence of acylsugars as a defense system to reduce whitefly oviposition and development in the field and most importantly to limit TYLCV infection by whiteflies in the field up to at least 51 days after planting. Our observation of the importance of the amounts of acylsugars to reduce whitefly oviposition and nymph survival is consistent with earlier studies [42, 45–47, 50–54, 70–73, 78–81], most of which were conducted with applications of purified acylsugars or on plants in controlled environments. Leckie et al [54] demonstrated increased control of whiteflies through *in planta* production of acylsugars using similar germplasm in field cages with source plants and non-viruliferous whiteflies and did not evaluate impact of acylsugar chemistries. Rodríguez-López et al [70] observed that the primary and secondary spread of TYLCV by viruliferous whiteflies in greenhouse studies to their acylsugar-accumulating tomato line decreased and was dependent on the time of year and concluded that acylsugar-mediated resistance in the lines tested was optimal in warm and dry growing regions and not likely to provide adequate virus protection for younger plant stages. Setiawati et al [79] evaluated several tomato varieties in a field trial and observed variation in whitefly abundance that was correlated with trichome abundance, but they did not evaluate the amount of acylsugars accumulated or acylsugar chemistry. Marchant et al [72] evaluated some of the same lines used in our study using lab assays, finding negative impacts of *in planta* produced acylsugars on whiteflies and a negative correlation between acylsugars and whitefly acquisition as well as transmission of TYLCV. These prior studies illustrate the potential impact of acylsugars on mediating resistance to whiteflies and their transmitted viruses, and the need for field trials validating the impact of *in planta* produced acylsugars.

Our study evaluated the impact of endogenous acylsugar production in tomato entries derived from *S*. *pennellii* [64] on the incidence of TYLCV in the field, starting with young plants. Notably, in comparison to the commercial tomato controls, whitefly egg and nymph abundance was decreased on the grouping of entries with higher amounts of acylsugars, particularly toward the end of the season, and decreased TYLCV infection at 51 DAP was observed in acylsugar accumulating tomato entries individually and in low, medium, and high acylsugar groupings. The strong association between increased acylsugar accumulation and decreased abundance of whitefly nymphs and TYLCV incidence at 51 DAP demonstrates clearly that the acylsugar defense system, as introgressed into the germplasm evaluated in this study, is effective in young and mature field-grown plants. While we cannot extrapolate incidence of TYLCV beyond 51 DAP, the reduced incidence of TYLCV up to that point is beyond the mid-point of the season when considerable fruit would have already been set. Therefore, the degree of reduction in TYLCV observed could have a profound economic impact because defending tomatoes against early infection by TYLCV is essential to preserve yield potential. Our results were obtained without any insecticide sprays and in the presence of numerous susceptible tomato entries. It is possible that even greater control is achievable if plants accumulating acylsugars are grown separately from whitefly susceptible varieties and if a supplemental IPM strategy is employed to help control whiteflies.

We evaluated variation among acylsugars accumulated by these entries in three ways: 1) the total amount of acylsugars accumulated, 2) the amount and relative proportion of acylsucroses and acylglucoses, and 3) the fatty acid profile. Our data revealed substantial variation in acylsugar amount across entries and strongly support an impact of acylsugar amount to reduce whitefly egg and nymph abundance as well as TYCLV incidence at 51 DAP. While the total amount of acylsugars accumulated were associated with resistance metrics, we did not observe an impact of the relative proportion of acylsucroses vs acylglucoses on whitefly egg or nymph abundance, or TYLCV incidence. When focusing on the entries that accumulated significant amounts of acylsugars, our data also revealed that the observed variation in the acylsugar fatty acid profile was more explanatory than that of acylsugar level. Specifically, the AIC analysis and our data support association of variation in specific acylsugar fatty acids (ai-C5, n-C10, and n-C12) with whitefly egg/nymph abundance and TYLCV incidence and may suggest interactions between fatty acid profile and acylsugar amount help moderate resistance. The observed association of fatty acids with resistance involved both the amount and relative proportion of particular fatty acids, suggesting that both aspects of the fatty acid profile are influential in mediating resistance. It is possible that the fatty acid sidechains of the acylsugars could differentially interact with and affect insects and the absolute amount of a particular acylsugar fatty acid(s) or the relative amount of a particular acylsugar fatty acid(s), or some combination of the two could help mediate deterrence/survival of whiteflies and a reduction in TYLCV.

Our experiments did not measure a cause-and-effect relationship between specific fatty acids and whitefly egg, nymph abundance and TYLCV incidence; however, our findings, particularly the AIC modeling, are important for a couple major reasons. 1) Minimum-AIC modeling identified several acylsugar fatty acids that are significantly associated with whitefly egg, nymph and TYLCV data after accounting for variation due to amount of acylsugar and the different whitefly and TYLCV pressures in each experiment. The abundance of individual acylsugar fatty acids can be manipulated through breeding [68, 69], and while specific acylsugar fatty acids and the significance of each one is difficult to ascertain in experiments like this, these data suggest the profile of acylsugar fatty acids matters and could interact with acylsugar amounts to impact whitefly egg, nymph abundance and TYLCV incidence at 51 DAP. 2) When the same methodology is applied to different experiments and systems, broader patterns can emerge and illuminate biological significance. Similar approaches by Ben-Mahmoud et al [82–84] indicated some acylsugar fatty acids identified in our study were also associated with western flower thrips (*Frankliniella occidentalis* Pergande) oviposition and incidence of *Tomato spotted wilt tospovirus*. The acylsugar fatty acid, n-C12 was previously associated with decreased western flower thrips oviposition on tomato [82, 83] and associated in our data with decreased whitefly oviposition. Similarly, n-C10 was previously associated with a decreased probability of *Tomato Spotted Wilt* incidence [83, 84]; in this experiment n-C10 was associated with a decrease in TYLCV incidence. These similarities could be coincidental, but further studies utilizing similar methodology will help clarify the involvement of these fatty acids in resistance. In particular our analysis suggests that the ai-C5, n-C10, and n-C12 acylsugar fatty acids are associated with whitefly egg and nymph abundance and TYLCV incidence up to at least 51 DAP. More targeted experiments are warranted and could help elucidate the impact of these acylsugar fatty acids in the context of resistance to insects and their transmitted viruses.

While we observed a general association of increased acylsugar accumulation with decreased abundance of whitefly eggs, variation for abundance of eggs within and across seasons was not always significant. There are a couple explanations for this observation. First, later in the season, some entries, particularly the tomato controls, were already dying or severely infected with TYLCV and could have been less acceptable to females for oviposition. The control plants were also more determinant in growth habit which could reduce quality of foliage toward end of season. Second, low whitefly pressure in two of the experiments and inherent variability in measuring oviposition made it more difficult to detect statistical differences.

We observed a clearer effect of acylsugars to reduce nymphs versus eggs when comparing the acylsugar amount groupings of entries. While it is certainly true that a reduction in oviposition should also lead to lower nymph counts, all entries sustained significant oviposition and the clearer reduction of nymphs attributed to acylsugar presence could be a combination of both antixenosis and antibiosis; this study did not attempt to separate these two effects. Further studies into the relative impact of antixenosis versus antibiosis from in-planta produced acylsugars to reduce whitefly populations are warranted.

There are three possible mechanisms to explain the general association of increased acylsugar accumulation with decreased abundance of whitefly eggs and nymphs and a decreased incidence of TYLCV at 51 DAP. One mechanism could be that observed by Rodríguez-López in two studies [70, 80] where whiteflies were deterred from landing and settling on acylsugar-accumulating plants compared to plants of a near-isogenic control with only trace-accumulation of acylsugars. The decision to oviposit eggs is an important choice for whitefly females and they follow a series of steps to identify a suitable host plant; in a non-hospitable environment this series of steps can be interrupted and as a result fewer or no eggs could be deposited [81]. A second related mechanism is that the density of type IV trichomes, predominantly associated with acylsugar production, is often higher on the abaxial leaflet surface [80]. Given a choice, whitefly adults and nymphs are predominantly found on the abaxial side of a leaflet [80, 85]; however, on an acylsugar-accumulating tomato, this preference is interrupted, and a significant number of the whiteflies that settle on an acylsugar tomato plant are found on the adaxial side of a leaflet [72, 80]; this could also affect oviposition. Additionally, survival of the largely sessile nymphs [86] on the adaxial leaf surface would likely be decreased due to increased likelihood of predation. The reduced egg and nymph abundance on tomato entries with higher amounts of acylsugars is, therefore, likely associated with the density of type IV trichomes. A third mechanism is that acylsugar-accumulation is associated with changes in whitefly feeding and probing behavior. Electrical penetration graph work by Rodriguez-Lopez et al [70, 80] demonstrated that on acylsugar-accumulating tomato leaflets whiteflies spend more time in non-probing activities and have a reduced ability to initiate probing. Moreover, Rodriguez-Lopez et al also show that whiteflies access the phloem less efficiently when feeding on acylsugar-accumulating tomato leaflets. The results of Marchant et al. [72] also support the role of acylsugars in reducing the likelihood that whiteflies acquire as well as transmit TYLCV. As probing and access to the phloem are integral to successful transmission of TYLCV by whiteflies, the behavioral changes associated with acylsugar-accumulation could have contributed to the decreased incidence of TYLCV in acylsugar-accumulating entries in our study.

Contrary to other analyses, the AIC analysis indicated a slight positive association between amount of acylsugar and TYLCV incidence. There are a couple explanations for this association. First, the AIC analysis only included data from entries with sufficient accumulation of acylsugars; the two standard tomato entries did not have sufficient accumulation of acylsugars to yield reliable measurement of acylsugar fatty acid profile, and so could not be considered. Second, it is possible that in some entries the types of acylsugars and acylsugar fatty acids that are associated with decreased incidence of TYLCV are at lower amounts/proportions than in other entries. Specifically, the n-C10 fatty acids, which are negatively correlated with TYLCV incidence, are present to a greater degree in the acylsugars of several entries with lower total amounts of accumulated acylsugars, therefore possibly creating a slight association of higher amount of acylsugar with increased incidence of TYLCV.

In summary, this study supports the utility of the acylsugar defense system in tomato to help control whiteflies and incidence of whitefly-transmitted TYLCV and contributes to the growing body of literature concerning the pursuit of sustainable, cost effective and safe methods to control insect pests of tomato, like whiteflies and their vectored viruses, and helps inform breeding targets to enhance the stability of tomato production worldwide.

## Supporting information

S1 Fig Acylsugar backbone profile of entries. Total accumulation amount of acylsugars of each entry averaged across samples and experiments. Acylsugars were determined to have either a sucrose (acylsucrose) or glucose (acylglucose) backbone.
(TIF)

S2 Fig Relative abundance of acylsugar fatty acids in profile of entries. Acylsugar fatty acids that constitute more than 1% of the total fatty acid profile of at least one entry were included in the analysis. The cumulative contributions of each acylsugar fatty acid to the total fatty acid profile of a given entry sum to 100%.
(TIF)

S3 Fig Absolute abundance of acylsugar fatty acids in profile of entries. Acylsugar fatty acids that constitute more than 1% of the total fatty acid profile of at least one entry were included in the analysis. Amounts of acylsugar fatty acids are presented as umol g^-1^ dry leaf weight.
(TIF)

S4 Fig Whitefly egg counts per 10 leaflets across acylsugar amount groupings Spring 2014. Number of whitefly eggs in Spring 2014 trial for each acylsugar amount grouping of entries. Four replicates pooled for each entry at each count date and averaged across entries within each acylsugar amount grouping at each count date. For each count date, acylsugar amount groupings of entries not connected by the same letter are significantly different (a = 0.05). Error bars represent one standard error of the mean.
(TIF)

S5 Fig Whitefly egg counts per 10 leaflets across acylsugar amount groupings Fall 2014. Number of whitefly eggs in Fall 2014 trial for each acylsugar amount grouping of entries. Four replicates pooled for each entry at each count date and averaged across entries within each acylsugar amount grouping at each count date. For each count date, acylsugar amount groupings of entries not connected by the same letter are significantly different (a = 0.05). Error bars represent one standard error of the mean.
(TIF)

S6 Fig Whitefly egg counts per 10 leaflets across acylsugar amount groupings Spring 2015. Number of whitefly eggs in Spring 2015 trial for each acylsugar amount grouping of entries. Four replicates pooled for each entry at each count date and averaged across entries within each acylsugar amount grouping at each count date. For each count date, acylsugar amount groupings of entries not connected by the same letter are significantly different (a = 0.05). Error bars represent one standard error of the mean.
(TIF)

S1 File All supporting data. (XLSX)

S1 Table Pedigrees or category of all entries included in field trials. (DOCX)

S2 Table AIC independent variables selected to model abundance of whitefly eggs. (DOCX)

S3 Table AIC independent variables selected to model abundance of whitefly nymphs. (DOCX)

S4 Table AIC independent variables selected to model incidence of TYLCV. (DOCX)
