# Supplementary figures and images for: The amount and chemistry of acylsugars affects sweetpotato whitefly (Bemisia tabaci) oviposition and development, and tomato yellow leaf curl virus incidence, in field grown tomato plants
Source: PLoS One. 2023 Nov 27;18(11):e0275112. doi: 10.1371/journal.pone.0275112 (PMC10681267; doi:10.1371/journal.pone.0275112)

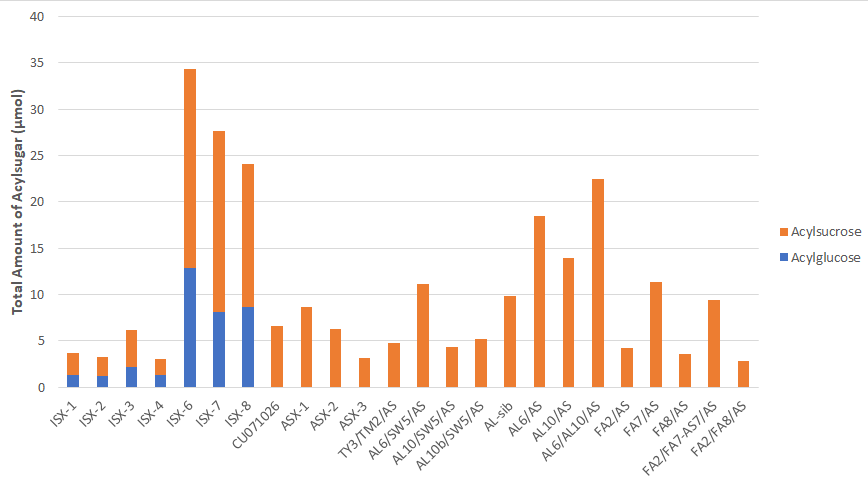

Supplement: S1 Fig — Total accumulation amount of acylsugars of each entry averaged across samples and experiments. Acylsugars were determined to have either a sucrose (acylsucrose) or glucose (acylglucose) backbone. (TIF) [file pone.0275112.s001.tif]

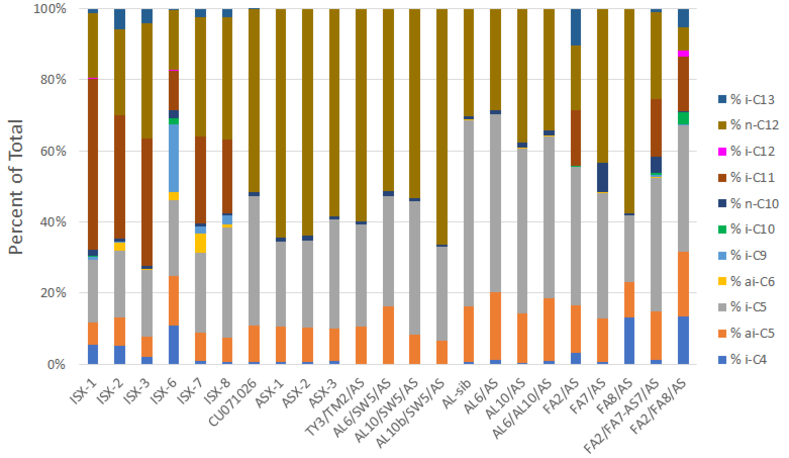

Supplement: S2 Fig — Acylsugar fatty acids that constitute more than 1% of the total fatty acid profile of at least one entry were included in the analysis. The cumulative contributions of each acylsugar fatty acid to the total fatty acid profile of a given entry sum to 100%. (TIF) [file pone.0275112.s002.tif]

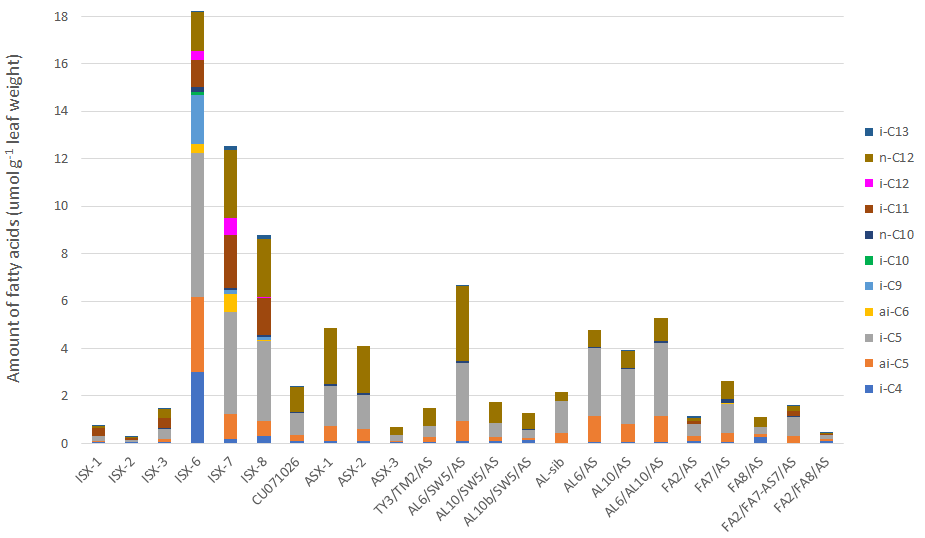

Supplement: S3 Fig — Acylsugar fatty acids that constitute more than 1% of the total fatty acid profile of at least one entry were included in the analysis. Amounts of acylsugar fatty acids are presented as umol g-1 dry leaf weight. (TIF) [file pone.0275112.s003.tif]

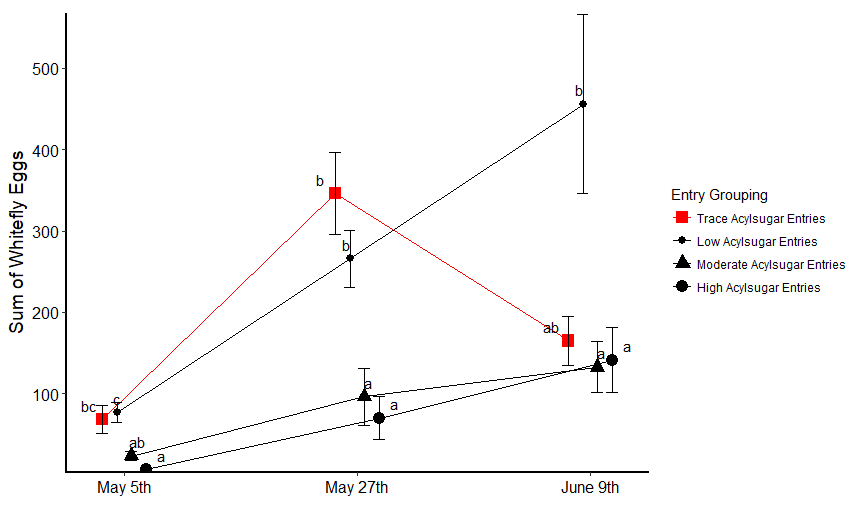

Supplement: S4 Fig — Number of whitefly eggs in Spring 2014 trial for each acylsugar amount grouping of entries. Four replicates pooled for each entry at each count date and averaged across entries within each acylsugar amount grouping at each count date. For each count date, acylsugar amount groupings of entries not connected by the same letter are significantly different (a = 0.05). Error bars represent one standard error of the mean. (TIF) [file pone.0275112.s004.tif]

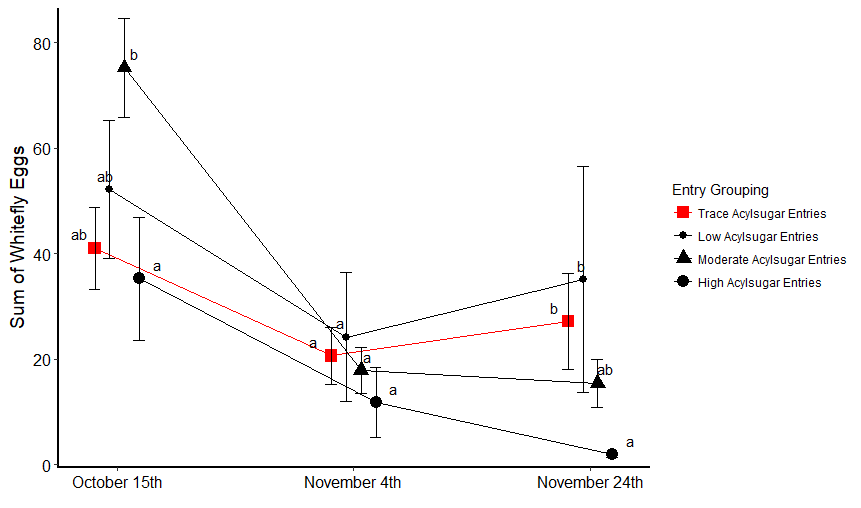

Supplement: S5 Fig — Number of whitefly eggs in Fall 2014 trial for each acylsugar amount grouping of entries. Four replicates pooled for each entry at each count date and averaged across entries within each acylsugar amount grouping at each count date. For each count date, acylsugar amount groupings of entries not connected by the same letter are significantly different (a = 0.05). Error bars represent one standard error of the mean. (TIF) [file pone.0275112.s005.tif]

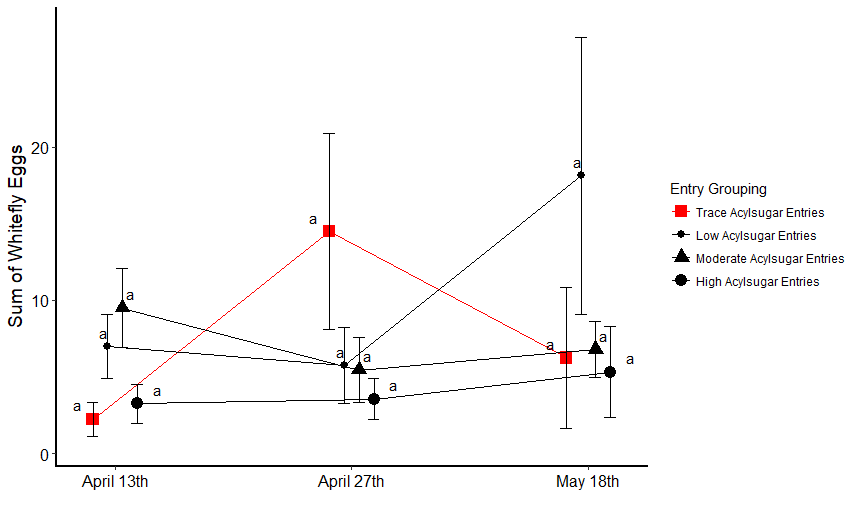

Supplement: S6 Fig — Number of whitefly eggs in Spring 2015 trial for each acylsugar amount grouping of entries. Four replicates pooled for each entry at each count date and averaged across entries within each acylsugar amount grouping at each count date. For each count date, acylsugar amount groupings of entries not connected by the same letter are significantly different (a = 0.05). Error bars represent one standard error of the mean. (TIF) [file pone.0275112.s006.tif]
