# Supplementary material for: The amount and chemistry of acylsugars affects sweetpotato whitefly (Bemisia tabaci) oviposition and development, and tomato yellow leaf curl virus incidence, in field grown tomato plants
Source: PLoS One. 2023 Nov 27;18(11):e0275112. doi: 10.1371/journal.pone.0275112 (PMC10681267; doi:10.1371/journal.pone.0275112)
Supplement: S1 Table — (DOCX) [file pone.0275112.s008.docx]

| Entry | Pedigree^a^ |
| --- | --- |
| ISX-1 | NC33EB1 X S. pennellii LA716 |
| ISX-2 | NC33EB1 X S. pennellii LA1732 |
| ISX-3 | NC33EB1 X S. pennellii LA1376 |
| ISX-4 | NC33EB1 X S. pennellii LA2560 |
| ISX-6 | CU071026 X S. pennellii LA1732 |
| ISX-7 | CU071026 X S. pennellii LA1376 |
| ISX-8 | CU071026 X S. pennellii LA2560 |

**S1 Table. Pedigrees of the interspecific entries included in the Spring 2014 and Fall 2014 field trials**

^a^ NC33EB1 is an inbred fresh market tomato with only trace acylsugar accumulation
