# Supplementary material for: The amount and chemistry of acylsugars affects sweetpotato whitefly (Bemisia tabaci) oviposition and development, and tomato yellow leaf curl virus incidence, in field grown tomato plants
Source: PLoS One. 2023 Nov 27;18(11):e0275112. doi: 10.1371/journal.pone.0275112 (PMC10681267; doi:10.1371/journal.pone.0275112)
Supplement: S2 Table — (DOCX) [file pone.0275112.s009.docx]

| **S2 Table. AIC independent variables selected to model abundance of whitefly eggs** | | | | | | |
| --- | --- | --- | --- | --- | --- | --- |
| Partial Regression Coefficients | | | | Analysis of Covariance (Type III Tests) | | |
| Parameter | Estimate | Std. Error | t value | Sum of Squares | df | P-value |
| Experiment | - | - | - | 105.57 | 2 | <0.001 |
| Acylsugar amount^a^ | -0.011 | 0.013 | -0.902 | 1.57 | 1 | 0.368 |
| Percent ai-C5^b^ | -0.094 | 0.030 | -3.168 | 19.36 | 1 | <0.001 |
| Umol n-C12^c^ | -0.433 | 0.075 | -5.781 | 64.44 | 1 | 0.002 |

^a^ Average amount of total acylsugars g^-1^ leaf weight across replicates for a given entry

^b^ Average percent of ai-C5 (antiso branched chain 5 carbon fatty acid) out of total fatty acids present across replicated samples for a given entry

^c^ Average amount of n-C12 (straight chain 12 carbon fatty acids) g^-1^ leaf weight across replicates for a given entry
