# Supplementary material for: The amount and chemistry of acylsugars affects sweetpotato whitefly (Bemisia tabaci) oviposition and development, and tomato yellow leaf curl virus incidence, in field grown tomato plants
Source: PLoS One. 2023 Nov 27;18(11):e0275112. doi: 10.1371/journal.pone.0275112 (PMC10681267; doi:10.1371/journal.pone.0275112)
Supplement: S4 Table — (DOCX) [file pone.0275112.s011.docx]

| **S4 Table. AIC independent variables selected to model incidence of TYLCV** | | | | | |
| --- | --- | --- | --- | --- | --- |
| Partial Regression Coefficients | | | Analysis of Deviance (Type III Tests) | | |
| Parameter | Estimate | Std. Error | Likelihood ꭓ^2^ | df | P-value |
| Intercept | -1.569 | 0.253 | - | - | - |
| Experiment | - | - | 106.739 | 2 | <0.001 |
| Acylsugar amount | 0.017 | 0.018 | 0.868 | 1 | 0.352 |
| Percent n-C10^a^ | -0.455 | 0.181 | 11.452 | 1 | <0.001 |

^a^ Average percent of fatty acid out of total fatty acids present across replicated GC-MS samples for a given entry
